# Supplementary figures and images for: Tin prefiltration in computed tomography does not significantly alter radiation-induced gene expression and DNA double-strand break formation
Source: PLoS One. 2024 Dec 20;19(12):e0315808. doi: 10.1371/journal.pone.0315808 (PMC11661594; doi:10.1371/journal.pone.0315808)

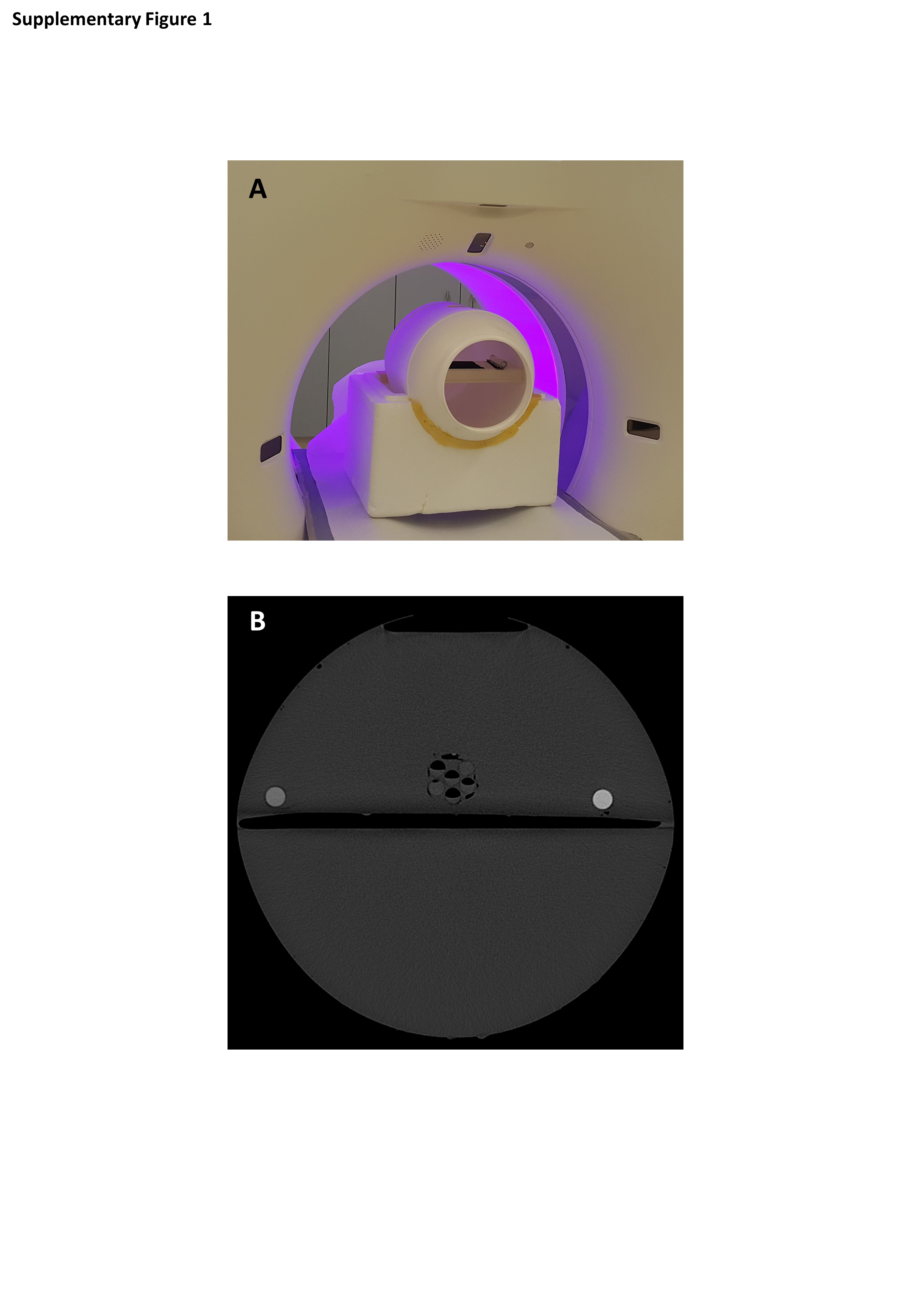

Supplement: S1 Fig — Picture of the 32-cm water phantom within the CT scanner (A) and CT-image (B) of the same water phantom, filled with 37°C tempered water and ETDA-blood tubes positioned in the center. The elongated light gray line measuring a few mm directly above the blood samples is the TLD positioned next to the blood samples. Two additional tubes filled with different contrast media concentrations are positioned on the side, which were not used for this study. Abbreviations: EDTA = ethylenediaminetetraacetic acid, TLD = thermoluminescent dosimeter. (TIF) [file pone.0315808.s001.tif]

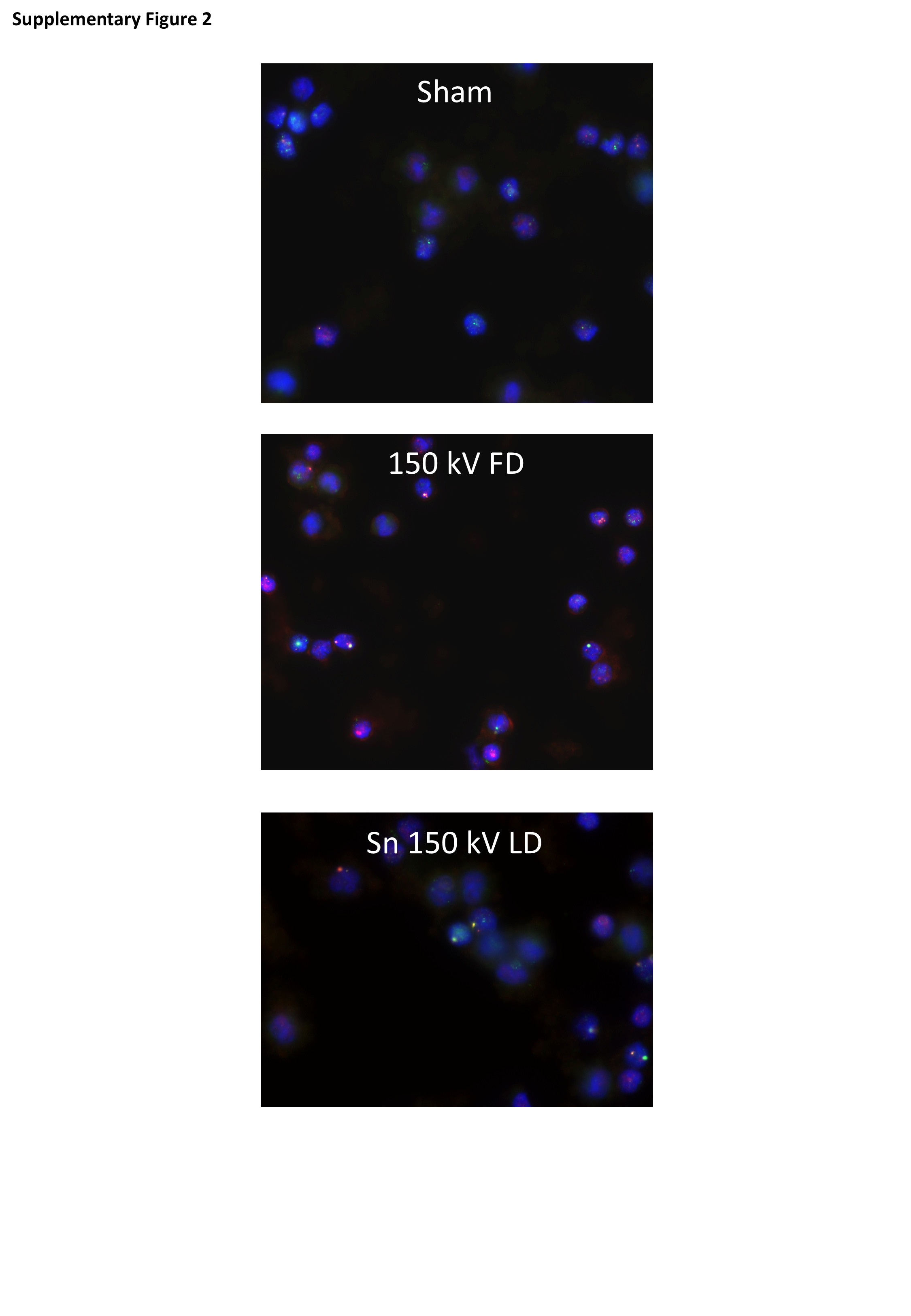

Supplement: S2 Fig — Example images show 20–21 peripheral blood cells (blue) per image with visualisation of γH2AX (green), 53BP1 (red) and co-localizing γ-H2AX + 53BP1 (yellow) double-strand breaks (DSB). There is an increase in DNA DSBs in X-irradiated samples. Abbreviations: FD = full dose, kV = kilovolt, LD = low dose, Sn = tin prefilter. (TIF) [file pone.0315808.s002.tif]

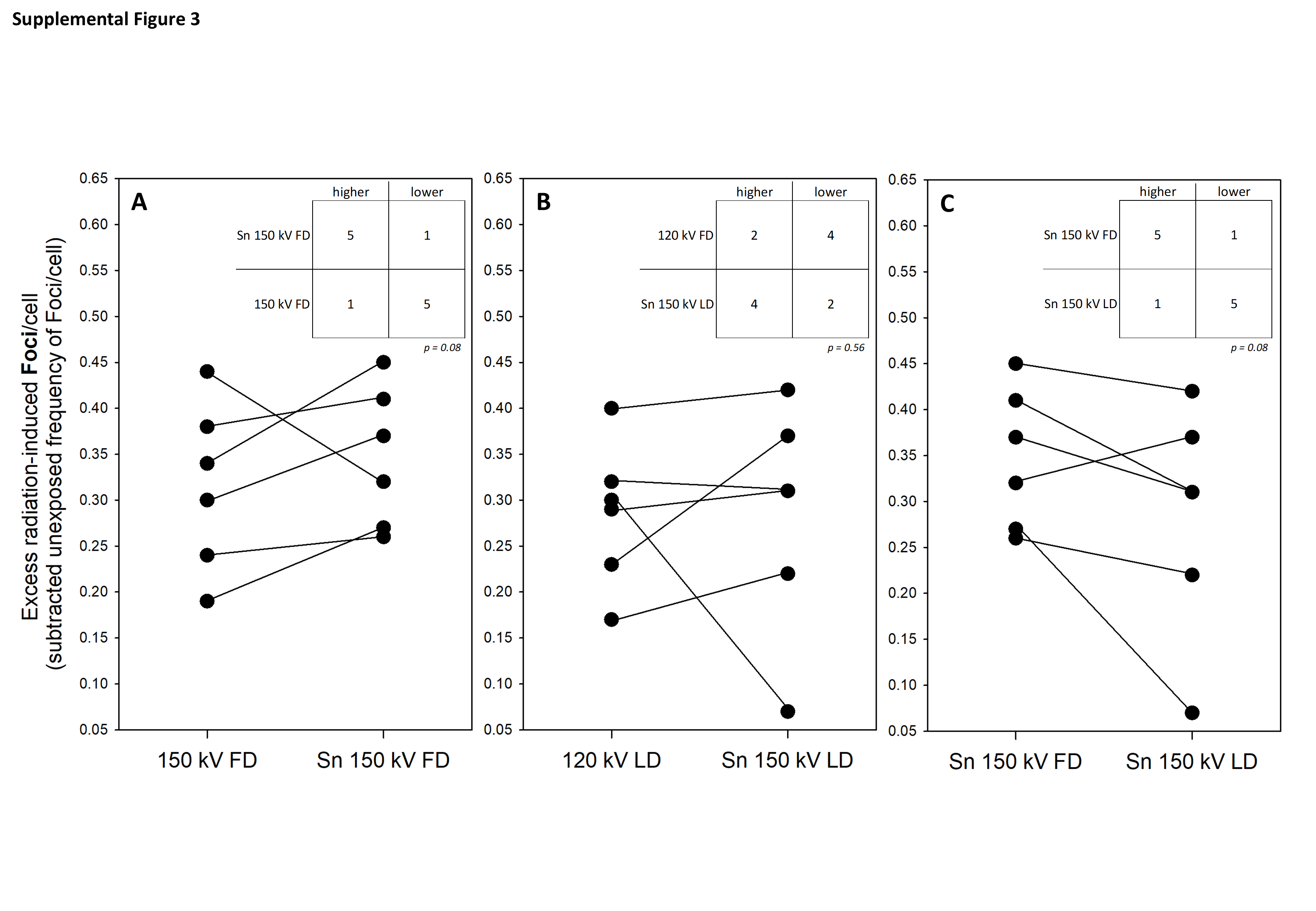

Supplement: S3 Fig — Graphical (connected scatter plot) and tabular (chi-square table) representation of the frequency distribution of higher resp. lower excess radiation-induced γH2AX Foci for the following research questions: A) 150 kV FD vs. Sn 150 kV FD, B) 120 kV FD vs. Sn 150 kV LD and C) Sn 150 kV FD vs. Sn 150 kV LD. The symbols reflect the mean values of excess radiation-induced foci per category. The line connects the excess radiation-induced Foci results of the same donor for both compared groups. Abbreviations: FD = full dose, kV = kilovolt, LD = low dose, Sn = tin prefilter. (TIF) [file pone.0315808.s003.tif]

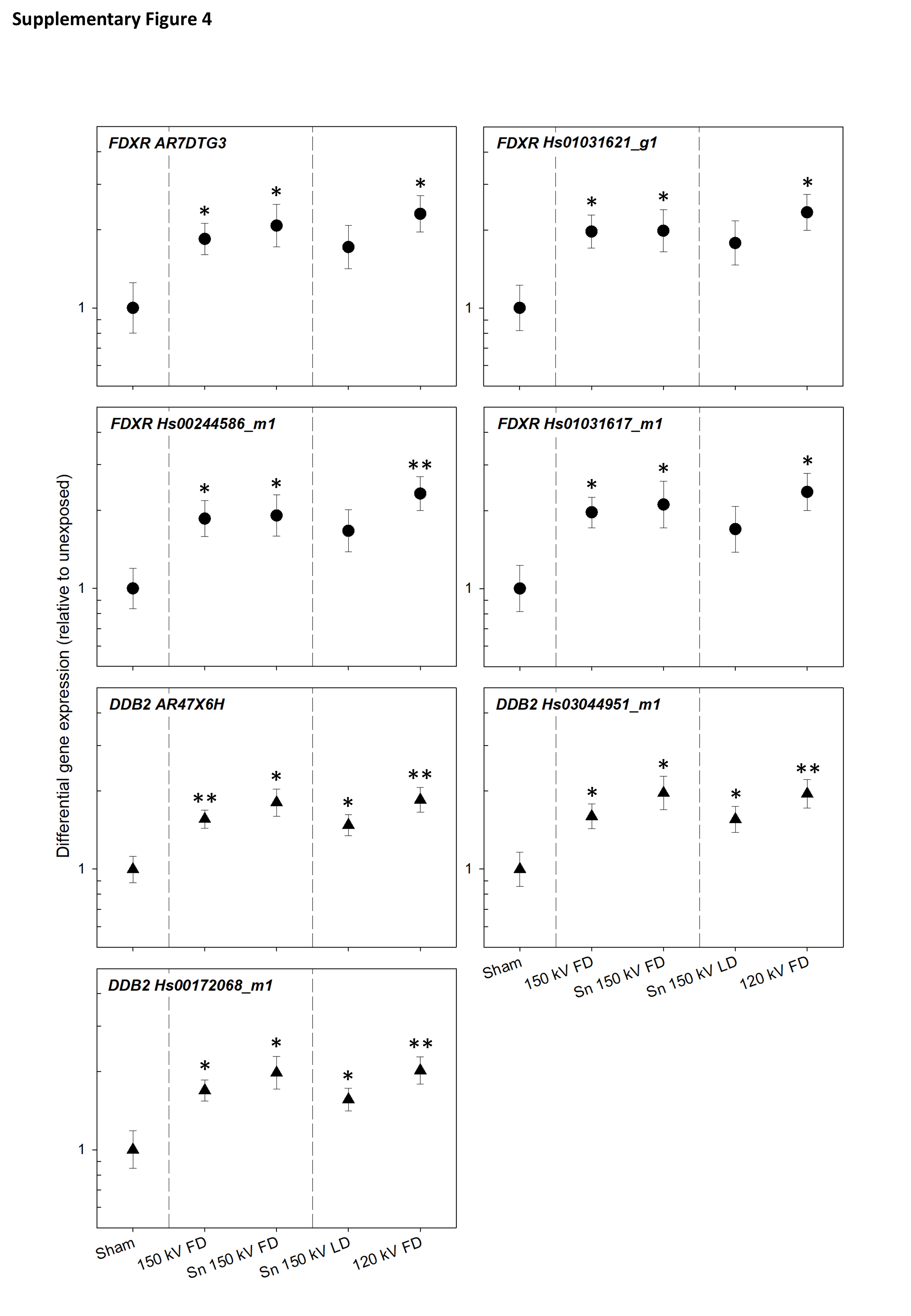

Supplement: S4 Fig — These Taq-Man assays were previously identified as most suitable for biodosimetry purposes [28]. The symbols reflect mean values, and error bars represent the standard error of the mean. P-values <0.05–0.01 and <0.01–0.001 are marked with one or two asterisks and refer to significant differences relative to unexposed values. Abbreviations: FD = full dose, kV = kilovolt, LD = low dose, Sn = tin prefilter. (TIF) [file pone.0315808.s004.tif]
