# Supplementary material for: Tin prefiltration in computed tomography does not significantly alter radiation-induced gene expression and DNA double-strand break formation
Source: PLoS One. 2024 Dec 20;19(12):e0315808. doi: 10.1371/journal.pone.0315808 (PMC11661594; doi:10.1371/journal.pone.0315808)
Supplement: Attachment — Submitted filename: Response to Reviewers_SIS_MA_V2.pdf [file pone.0315808.s008.pdf]

Responses to journal requirements/to the reviewers

Thank you for considering our manuscript. Our answers to your requests are listed point-by-point below.

Journal requirements:

When submitting your revision, we need you to address these additional requirements.

1. Please ensure that your manuscript meets PLOS ONE's style requirements, including those for file naming. The PLOS ONE style templates can be found at

https://journals.plos.org/plosone/s/file?id=wjVg/PLOSOne_formatting_sample_main_body.pdf and

https://journals.plos.org/plosone/s/file?id=ba62/PLOSOne_formatting_sample_title_authors_affiliations.pdf

Response; thank you for this remark. We have changed the name of the manuscript files accordingly ("Revised Manuscript with Track Changes" and "Manuscript"). We believe that the file names for the figures (Fig1, Fig2, etc.) and the file names for the supporting information (S1_Fig, S1_Table, etc.) were correct according to the journals style requirements. If incorrect style requirements are still present in the revised submission, we would be grateful for specific information and we will be happy to correct them.

2. We note that you have included the phrase "data not shown" in your manuscript. Unfortunately, this does not meet our data sharing requirements. PLOS does not permit references to inaccessible data. We require that authors provide all relevant data within the paper, Supporting Information files, or in an acceptable, public repository. Please add a citation to support this phrase or upload the data that corresponds with these findings to a stable repository (such as Figshare or Dryad) and provide and URLs, DOIs, or accession numbers that may be used to access these data. Or, if the data are not a core part of the research being presented in your study, we ask that you remove the phrase that refers to these data.

Response; we have added a supplementary figure (S3 Fig) showing the data.

3. Please review your reference list to ensure that it is complete and correct. If you have cited papers that have been retracted, please include the rationale for doing so in the manuscript text, or remove these references and replace them with relevant current references. Any changes to the reference list should be mentioned in the rebuttal letter that accompanies your revised manuscript. If you need to cite a retracted article, indicate the article's retracted status in the References list and also include a citation and full reference for the retraction notice.

Response; We have reviewed our reference list and believe it to be complete and accurate.

Reviewers' comments:

Reviewer's Responses to Questions

1. Is the manuscript technically sound, and do the data support the conclusions?

The manuscript must describe a technically sound piece of scientific research with data that supports the conclusions. Experiments must have been conducted rigorously, with appropriate controls, replication, and sample sizes. The conclusions must be drawn appropriately based on the data presented.

Reviewer #1: Yes

Reviewer #2: Yes

Response; thank you for this assessment.

2. Has the statistical analysis been performed appropriately and rigorously?

Reviewer #1: Yes

Reviewer #2: Yes

Response; thank you for this assessment.

3. Have the authors made all data underlying the findings in their manuscript fully available?

The PLOS Data policy requires authors to make all data underlying the findings described in their manuscript fully available without restriction, with rare exception (please refer to the Data Availability Statement in the manuscript PDF file). The data should be provided as part of the manuscript or its supporting information, or deposited to a public repository. For example, in addition to summary statistics, the data points behind means, medians and variance measures should be available. If there are restrictions on publicly sharing data—e.g. participant privacy or use of data from a third party—those must be specified.

Reviewer #1: Yes

Reviewer #2: Yes

Response; thank you for this assessment.

4. Is the manuscript presented in an intelligible fashion and written in standard English?

PLOS ONE does not copyedit accepted manuscripts, so the language in submitted articles must be clear, correct, and unambiguous. Any typographical or grammatical errors should be corrected at revision, so please note any specific errors here.

Reviewer #1: Yes

Reviewer #2: Yes

Response; thank you for this assessment.

5. Review Comments to the Author

Please use the space provided to explain your answers to the questions above. You may also include additional comments for the author, including concerns about dual publication, research ethics, or publication ethics. (Please upload your review as an attachment if it exceeds 20,000 characters)

Reviewer #1: General Comments

The manuscript titled "Tin prefiltration in computed tomography fails to alter radiation-induced gene expression and double-strand break formation" presents an interesting study on the molecular biological response to the tin prefilter technique in CT. The authors examine both radiation-induced double-strand break (DSB) formation and differential gene expression (DGE) in blood samples exposed to different CT protocols. The study is relevant in the context of reducing radiation dose without compromising image quality, and the results indicate that this technique does not significantly affect radiation-induced molecular changes when compared to standard CT protocols.

Response; we would like to thank the reviewer for the thorough and very helpful comments and for the overall positive feedback.

Specific Comments:

1. Abbreviations, Units, and Sentence Structure:

The entire manuscript requires a thorough revision to ensure consistent use of abbreviations, correct application of units in the biological endpoints measured, and the construction of complete sentences.

Response; thank you for this remark, we reviewed our manuscript and ensured a consistent use of abbreviations, the correct application of units in de biological endpoints and the construction of complete sentences. If we have overlooked any of the points you have mentioned in the manuscript, please let us know again in detail so that we can make the necessary corrections.

2. Image Quality Technique:

The description of image quality techniques should be more detailed and clearer. It is essential to specify whether the quality of the images was evaluated objectively or subjectively.

Response; thank you for this remark. We adjusted the corresponding methods section to: *"For this purpose, the image quality was assessed objectively. The standard deviation of the mean Houndsfield Unit value of a region of interest (ROI) in the air in the CT image was used as the image quality parameter (background noise). The radiation dose of the Sn 150 kV LD protocol was determined as follows: First, the image noise of the 120 kV FD protocol was measured with triplicate ROI measurements in CT images, then the radiation dose (mAs) of the Sn 150 kV protocol was lowered until triplicate ROI measurements in CT images at the same position showed the same image quality as the 120 kV FD protocol."* We hope this clarifies our approach.

3. Title Suggestion:

I recommend revising the title to one of the following options for clarity and impact:

- Tin prefiltration in computed tomography does not significantly alter radiation-induced gene expression and DNA double-strand breaks."

- Minimal effects of tin prefiltration on radiation-induced gene expression and DNA double-strand break formation in computed tomography."

Response; we have changed the title to "Tin prefiltration in computed tomography does not significantly alter radiation-induced gene expression and DNA double-strand break formation".

4. Abstract Revision (Page 3):

In the conclusion of the abstract, instead of "our ex vitro," it is better to write "the current ex-vivo study."

Response; we have changed the sentence accordingly.

5. miRNA Notation:

The first miRNA should be correctly noted as "MicroRNA-667."

Response; thank you for this remark. We think there is a misunderstanding. We analysed 667 different microRNAs. miR-667 was not among them. We changed the sentence accordingly: "Post-transcriptional measurements on altogether 667 different miRNA species using LDAs"

6. Dose Measurement Methodology:

The methodology section should clearly explain how the dose was measured or calculated, whether through dosimetry or other methods.

Response; the corresponding sentence in the Material and Methods section has been changed as follows: *"The applied radiation dose was measured with thermoluminescence dosimeters*

(TLD) (Karlsruhe Institute of Technology, Karlsruhe, Germany) in terms of kerma in water. The TLDs were placed next to the blood samples in the water phantom for each CT scan." Additionally, in the footnotes table 1 we added the following sentence: *"The asterisk marks the mean radiation dose measured with thermoluminescence dosimeters ± standard deviation (Kerma in water)."*

We hope this clarifies how the dose was measured.

7. Control Blood Samples:

The exclusion criteria for the healthy control blood samples should be specified. It would have been better to compare the same donor blood samples before and after irradiation. If controls excluded certain criteria such as smoking or chemotherapy, this should be clearly stated.

Response; thank you for this remark.

To clarify the inclusion criteria for the human donors we added the following sentence to the Material and Methods section: *"The following inclusion criteria apply to the healthy donors: age ≥ 18 years, no acute health problems, no previously known diseases of the haematopoietic system, signed written informed consent form, ability to give consent, naivety towards exposure to higher radiation doses (< 1 mSv/life year)."*

Also, we did compare the same donor blood samples before and after irradiation. Each donor gave blood into five 4.9 ml EDTA blood tubes. Per donor one EDTA blood tube was not irradiated and served as the reference (control) sample. Three EDTA tubes were irradiated with 20 mGy with 150 kV, Sn 150 kV or 120 kV and one EDTA blood tube was irradiated with 16.5 mGy with Sn 150 kV. The unirradiated control or reference sample was later used to calculate the differential gene expression of each irradiated sample (DGE = $2^{-\Delta\Delta Ct}$) per examined gene or to calculate the excess radiation-induced Foci by subtracting the counted foci per cell of the respective unexposed sample from the counted foci per cell value of the irradiated sample of each donor.

To clarify that, we adjusted the following sentence: *"For each donor the ratio (DGE) relative to the unexposed sample was determined by the ΔΔCt approach (DGE = $2^{-\Delta\Delta Ct}$)."*

8. Discussion on DGE Values:

Although the paper concludes that tin prefiltration does not alter radiation-induced DGE or DSB formation, it also notes a significant tendency toward lower DGE values for the Sn 150 kV low-dose protocol. This finding needs clarification. The discussion should be expanded to better explain the biological implications of this trend toward lower DGE values. Additionally, it should be explicitly stated whether these changes, though not statistically significant, could still hold clinical relevance.

Response; thank you for this important remark. We have expanded the discussion an added the following sentences: "*Due to the non-significant changes in the direct group comparisons, these results of the frequency distribution should not be overinterpreted, so that the overall*

conclusion of this study, that the tin prefilter does not lead to any relevant change in the analyzed biological endpoints, remains valid. The results of the frequency distribution are nevertheless worth mentioning, as they show the protective, expected effect of the tin prefilter technique due to the dose reduction while maintaining image quality."

Reviewer #2:

The authors have presented a careful study of an assessment of the tin pre-filtration technique for CT scanning. This technique has the ability to reduce radiation exposure during CT scans by eliminating the lower energy radiation from the scanners which gets absorbed by a thin layer of tin (Sn), allowing only the higher energy radiation. This is best suited for deep and high contrast imaging. A thorough evaluation of the possible protective effect of this technology is significant and this reviewer believes it has been done carefully, and with suitable sampling (at least for the DNA DSB marker yH2AX), and with appropriate assays. The authors chose to concentrate on the DNA damage aspects of CT scanning. This reviewer believes this study was straightforward, and presents reliable data that is easily decipherable and would be of value to the field. Care was also taken to control for dose levels, and several iterations were used for a full comparison. It was also commendable that only yH2AX foci co-localized with 53BP1 were counted, and yH2AX is known to mark non-DSB lesions as well. The number of patient/samples (6) could have been higher, which might have helped reducing variability in the transcriptomics and miRNA (but that was acknowledge by the authors).

Response; we would like to thank the reviewer for the thorough and very helpful comments and for the overall positive feedback.

some minor edits:

line 242 Fig 2. yH2AX foci: the scoring and tabulating methodologies for this assay are sound. according to the method to score these the number of foci per cell of unexposed is subtracted from the exposed. The resulting increase in foci count is not that high, it is less than 1 foci per cell, but nevertheless significant. This reviewer would be heartened to see some example images. The increase in foci might not be perceivable, but showing example imaging (even if in the supplement) would boost confidence in the technique.

Response; thank you for this remark. We think that it is an excellent idea to add a supplementary figure, showing some cells with DNA DSB foci for unexposed and exposed samples (S2 Fig). As outlined by the well-respected reviewer, the images primarily show the quality of our labelling technique. Due to the low difference in foci, it is difficult to provide images that easily distinguish irradiated from non-irradiated cells. Nevertheless, one can tend to recognise more foci in the images of the irradiated samples.

line 370-371

suggested change to

"can distinguish between whole-body and partial-body irradiation [33]."

Response; we changed the sentence accordingly.

line 374

suggested change to

"low dose irradiation has not been"

Response; thank you for this remark. We corrected the typo accordingly.

line 403 and 404

change deregulated to 'dysregulated'

Response; we changed 'deregulated' to 'dysregulated' throughout the manuscript, including line 403 and 404.

line 407

change genome to 'transcriptome'

Response; thank you for reading so carefully. You are correct, it should read 'transcriptome', and we have changed it accordingly.

line 415

suggested change to

"This is a limitation of this study, and which was attempted to be minimized by performing the trial scans."

Response; we changed the sentence to "This is a limitation of this study, which was attempted to be minimized by performing the trial scans".
