# Supplementary material for: Tin prefiltration in computed tomography does not significantly alter radiation-induced gene expression and DNA double-strand break formation
Source: PLoS One. 2024 Dec 20;19(12):e0315808. doi: 10.1371/journal.pone.0315808 (PMC11661594; doi:10.1371/journal.pone.0315808)
Supplement: Attachment — Submitted filename: General Comments.docx [file pone.0315808.s007.docx]

**General Comments**

The manuscript titled "*Tin prefiltration in computed tomography fails to alter radiation-induced gene expression and double-strand break formation*" presents an interesting study on the molecular biological response to the tin prefilter technique in CT. The authors examine both radiation-induced double-strand break (DSB) formation and differential gene expression (DGE) in blood samples exposed to different CT protocols. The study is relevant in the context of reducing radiation dose without compromising image quality, and the results indicate that this technique does not significantly affect radiation-induced molecular changes when compared to standard CT protocols.

**Specific Comments:**

1. **Abbreviations, Units, and Sentence Structure**:
   The entire manuscript requires a thorough revision to ensure consistent use of abbreviations, correct application of units in the biological endpoints measured, and the construction of complete sentences.
2. **Image Quality Technique**:
   The description of image quality techniques should be more detailed and clearer. It is essential to specify whether the quality of the images was evaluated objectively or subjectively.
3. **Title Suggestion**:
   I recommend revising the title to one of the following options for clarity and impact:

- Tin prefiltration in computed tomography does not significantly alter radiation-induced gene expression and DNA double-strand breaks."
- Minimal effects of tin prefiltration on radiation-induced gene expression and DNA double-strand break formation in computed tomography."

4. **Abstract Revision (Page 3)**:
   In the conclusion of the abstract, instead of "our ex vitro," it is better to write "the current ex-vivo study."
5. **miRNA Notation**:
   The first miRNA should be correctly noted as "MicroRNA-667."
6. **Dose Measurement Methodology**:
   The methodology section should clearly explain how the dose was measured or calculated, whether through dosimetry or other methods.
7. **Control Blood Samples**:
   The exclusion criteria for the healthy control blood samples should be specified. It would have been better to compare the same donor blood samples before and after irradiation. If controls excluded certain criteria such as smoking or chemotherapy, this should be clearly stated.
8. **Discussion on DGE Values**:
   Although the paper concludes that tin prefiltration does not alter radiation-induced DGE or DSB formation, it also notes a significant tendency toward lower DGE values for the Sn 150 kV low-dose protocol. This finding needs clarification. The discussion should be expanded to better explain the biological implications of this trend toward lower DGE values. Additionally, it should be explicitly stated whether these changes, though not statistically significant, could still hold clinical relevance.
